# LncRNA BDNF-AS is associated with the malignant status and regulates cell proliferation and apoptosis in osteosarcoma

**DOI:** 10.1042/BSR20181498

**Published:** 2018-11-16

**Authors:** Qiang Huang, Jiao Yang, Xin He, Shuyan Shi, Shuxing Xing

**Affiliations:** Department of Orthopaedics, Chengdu Fifth People’s Hospital, Chengdu 611130, Sichuan, China

**Keywords:** BDNF-AS, biomarker, large intervening non-coding RNA, osteosarcoma

## Abstract

Long non-coding RNA (LncRNA) brain-derived neurotrophic factor antisense (BDNF-AS) has been found to be down-regulated and function in a tumor suppressive role in human cancers. However, the expression status and function of BDNF-AS is still unknown in osteosarcoma (OS). In our study, BDNF-AS expression was found to be decreased in OS tissues and cells. Moreover, BDNF-AS low expression was correlated with advanced Enneking stage, large tumor size and poor prognosis in OS patients. The multivariate analysis suggested low expression of BDNF-AS was an independent unfavorable prognostic factor for overall survival in OS patients. The *in vitro* studies indicated that BDNF-AS overexpression inhibits OS cell proliferation and promotes cell apoptosis through regulating cleaved caspase-3. In conclusion, BDNF-AS serves as a tumor suppressive lncRNA in OS.

## Introduction

Osteosarcoma (OS) is the most common malignant bone tumor, and the major causes of cancer–associated mortality in children and young adults [1]. An updated cancer statistics suggested that 24000 new OS cases occurred and 17000 cases died from OS in China in 2014 [2]. OS is derived from primitive mesenchymal stem cells characterized by high malignancy, frequent recurrence, and distant metastasis [3]. Due to the development in chemotherapy combined with surgery and radiotherapy, the 5-year survival rate of OS has been plateaued with more than 60% [4]. However, the 5-year survival rate of advanced OS patients is only 27.4% [5]. Therefore, it is necessary for improving the prognosis of OS patients to illuminate the pathogenesis and progression of OS and identify novel biomarkers.

Long non-coding RNAs (lncRNAs) are a group of poorly conserved endogenous RNA molecules longer than 200 nts in length without ORFs [6]. Growing evidence suggested dysregulated expression of lncRNAs was involved in various cellular processes, such as cell proliferation, cell cycle, migration, invasion, and apoptosis [7]. Recently, several lncRNAs have been found to serve as tumor suppressors or promoters in OS carcinogenesis, such as GAS5 [8], SOX2-OT [9], ZEB1-AS1 [10], SNHG15 [11], XIST [12], NEAT1 [13] etc.

LncRNA brain-derived neurotrophicfactor antisense (BDNF-AS) was originally discovered as a natural non-coding antisense of neuronal transcriptional factor BDNF [14,15]. Subsequently, BDNF-AS has been found to be down-regulated and function in tumor suppressive role in lung cancer [16], prostate cancer [17], esophageal cancer [18], cervical cancer [19], and retinoblastoma [20]. However, the role of BDNF-AS is still unknown in OS. We suppose that BDNF-AS also acts as a tumor suppressor in the pathogenesis and progression of OS. Therefore, the purposes of present study were to investigate the expression pattern and clinical significance of BDNF-AS in OS patients and biological function and molecular mechanism of BDNF-AS in OS cells.

## Materials and methods

### Tissue samples

The present study was approved by the Ethics committee of Chengdu Fifth People’s Hospital. The cohort consisted of 114 OS samples and 35 paired non-cancerous samples. Written informed consent was obtained from all patients. All clinical tissues were frozen in liquid nitrogen immediately after surgical resection or biopsy and stored at −80°C. The relevant clinical and survival data were available for all cases. All the patients were primary OS patients without a history of other tumors and none of these patients underwent preoperative chemotherapy or radiotherapy.

### Cell culture

The cell lines (three OS cell lines Saos-2, MG-63, and HOS, and a human normal osteoblast cell line hFOB1.19) were obtained from Cell Bank of Type Culture Collection of Chinese Academy of Sciences (Shanghai, China). All cell lines were cultured in Roswell Park Memorial Institute (RPMI)-1640 medium (Gibco, Grand Island, NY, U.S.A.) containing 10% FBS (Gibco, Grand Island, NY, U.S.A.) at 37°C in a humidified atmosphere containing 5% CO_2_.

### RNA extraction and quantitative real-time PCR

Total RNAs were extracted from tissues or cells using RNAiso Plus (Takara, Dalian, China) following the manufacturer’s instructions. Real-time qPCR was performed at ABI 7500 system (Applied Systems, Foster City, CA, U.S.A.) using the SYBR Premix ExTaqII (Takara, Dalian, China) with the following primers. Real-time qPCR was performed at Roche LightCycler using the SYBR Premix ExTaqII (Takara, Dalian, China) with the following primers. The primers’ sequences were: BDNF-AS, forward primer, 5′-CCGTGAGAAGATCTCATTGGG-3′ and reverse primer, 5′-GGGTCACAAGTCACGTAGCA-3′; GAPDH, forward primer, 5′-CAGCCTCAAGATCATCAGCA-3′ and reverse primer, 5′-TGTGGTCATGAGTCCTTCCA-3′. All experiments were performed in triplicate and normalized to GAPDH.

### Plasmid transfection

The BDNF-AS overexpressing vector (named as BDNF-AS) and corresponding negative control vector (named as pcDNA) were designed and constructed by Ribobio (Guangzhou, China). Transfection of vectors were performed using Lipofectamine 3000 (Invitrogen, Carlsbad, CA, U.S.A.) according to the manufacturer’s guidelines.

### Cell proliferation assay

Cell proliferation was determined using the MTT assay. Briefly, 5 × 10^3^ cells were plated per well in 96-well plates. After 1, 3, and 5 days transfection, MTT solution dissolved in culture medium at a concentration of 5 mg/ml was added to each well. After 4 h of incubation, 100 μl of DMSO (Sigma–Aldrich, MI, U.S.A.) was added to solubilize MTT tetrazolium crystal and the optical density was at 590 nm using a microplate reader (Bio-Rad, Hercules, CA, U.S.A.).

### Cell colony formation assay

After transfection with BDNF-AS or pcDNA, 5 × 10^2^ cells OS cells were plated in each well of the six-well plate and cultured for 10 days. The colonies were then washed twice with PBS, fixed with 4% paraformaldehyde, and stained with 0.1% Crystal Violet. The number of colonies containing ≥50 cells was counted under a light microscope.

### Flow cytometry analysis of cell apoptosis

The apoptosis ratio was analyzed using the Annexin V-FITC Apoptosis Detection Kit (Keygen, Nanjing, China). At 48 h after transfection, OS cells were harvested and re-suspended in binding buffer containing Annexin V-FITC and PI according to the manufacturer’s guidelines. The stained cells were analyzed by flow cytometry. The percentages of apoptotic cells from each group were compared.

### Western blot

Total proteins were prepared from colorectal cells using RIPA buffer (Boster, Wuhan, China). The protein concentration was measured by BCA kit (Beyotime, Beijing, China). Equal quantities of protein were electrophoresed through SDS/PAGE (10% gel) to PVDF membranes (Millipore, MA, U.S.A.). The membranes were blocked and then incubated with antibodies against caspase-3, cleaved caspase-3 (Cell Signaling Technology, CA, U.S.A.) or β-actin (Beyotime, Beijing, China) overnight at 4°C. Then, the membranes were incubated with HPR–conjugated secondary antibodies (Cell Signaling Technology, CA, U.S.A.) for 2 h. The protein bands were visualized using chemiluminescence reagent kit (ECL, Beyotime, Beijing, China), analyzed by Quantity One Software (Bio-Rad, Hercules, CA, U.S.A.). β-actin was used as an endogenous protein for normalization.

### Statistical analysis

Statistical analyses were performed using SPSS 17.0 version. Chi-squared test was used to analyze the relationship between BDNF-AS expression and clinicopathological characteristics. The significance of differences between mean values was determined by Student’s independent sample *t*test. Survival data were analyzed using Kaplan–Meier method and log-rank test. Univariate and multivariate survival analyses were performed using the likelihood ratio test of the stratified Cox proportional hazards model. A *P*-value <0.05 was considered significant.

## Results

### Expression pattern of BDNF-AS in OS tissues and cells

**Figure 1:**
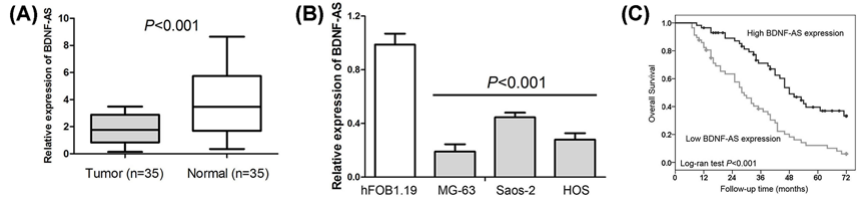
The expression status and prognostic value of BDNF-AS in OS (**A**) Low expression of BDNF-AS is observed in OS tissues compared with non-cancerous normal tissues. (**B**) OS cell lines MG-63, Saos-2, and HOS exhibit the lower level expression of BDNF-AS in comparison with human normal osteoblast cell line hFOB1.19. (**C**) Low BDNF-AS expression predicts unfavorable prognosis in OS patients.

To determine the expression status of BDNF-AS in OS tissue, the levels of BDNF-AS expression were detected in 35 pairs of OS tissues and non-cancerous normal tissues through quantitative real-time PCR (qRT-PCR). Compared with non-cancerous normal tissues, low expression of BDNF-AS was observed in OS tissues (*P*<0.001, Figure 1A). Moreover, levels of BDNF-AS expression were also determined in three OS cell lines MG-63, Saos-2, and HOS and a human normal osteoblast cell line hFOB1.19. We found OS cell lines exhibited a significantly lower level expression of BDNF-AS in comparison with human normal osteoblast cell line (*P*<0.001, Figure 1B).

### Reduced BDNF-AS expression is associated with the progression of OS patients

On the basis of the result that BDNF-AS was down-regulated in OS, we sought to explore the correlation of BDNF-AS expression with OS progression. Based on the median value of BDNF-AS expression, all OS patients were divided into high or low expression subgroup. The correlation between BDNF-AS expression and clinicopathological parameters of OS cases was analyzed and summarized in Table 1. BDNF-AS expression was not obviously correlated with gender (*P*=0.691), age (*P*=0.570), and histological grade (*P*=0.445). However, reduced BDNF-AS expression was found to be correlated with advanced Enneking stage (*P*=0.032) and large tumor size (*P*=0.020).

**Table 1:** Correlations between BDNF-AS expression and clinical parameters in OS patients

Parameters	*n*	Low expression	High expression	*P*
Age (years)				
≤18	49	26	23	0.570
>18	65	31	34	
Gender				
Female	38	18	20	0.691
Male	76	39	37	
Enneking stage				
I–II	41	15	26	0.032
II B–III	73	42	31	
Tumor size				
≤8 cm	53	21	32	0.020
>8 cm or discontinuous tumors	61	36	25	
Histological grade				
G1–G2	68	32	36	0.445
G3–G4	46	25	21	

### Reduced BDNF-AS expression is associated with unfavorable prognosis in OS patients

To understand the prognostic significance of BDNF-AS in OS cases, the relationship between BDNF-AS expression and overall survival were estimated in 114 OS patients. As shown in Figure 1C, the low expression of BDNF-AS group had a significantly poorer prognosis than the high expression of BDNF-AS group (*P*<0.001). Besides, the univariate Cox regression analysis suggested that Enneking stage (*P*<0.001), large tumor size (*P*<0.001), and BDNF-AS low expression (*P*<0.001) were unfavorable prognostic factors for overall survival in OS cases (Table 2). Furthermore, the multivariate analysis suggested Enneking stage (*P*=0.002) and BDNF-AS expression (*P*=0.007) were independent unfavorable prognostic factors for overall survival in OS cases (Table 2).

**Table 2:** Univariate and multivariate Cox regression of prognostic factors for overall survival in OS patients

Parameter	Univariate analysis			Multivariate analysis		
	HR	95% CI	*P*	HR	95% CI	*P*
Age (years) (≤18 compared with >18)	0.869	0.559–1.350	0.531			
Gender (Female compared with male)	1.365	0.845–2.205	0.204			
Enneking stage (I-II A compared with II B-III)	4.661	2.686–8.091	<0.001	3.610	1.627–8.010	0.002
Tumor size (≤8 compared with >8 cm or discontinuous tumors)	3.171	1.954–5.148	<0.001	1.060	0.538–2.088	0.866
Histological grade (G1–G2 compared with G3–G4)	1.288	0.822–2.018	0.270			
BDNF-AS expression (Low compared with high)	0.375	0.238–0.591	<0.001	0.524	0.327–0.838	0.007

Abbreviations: HR, hazard ratio; 95% CI, 95% confidence interval.

### BDNF-AS expression inhibits OS cell proliferation and promotes cell apoptosis through regulating cleaved caspase-3

**Figure 2:**
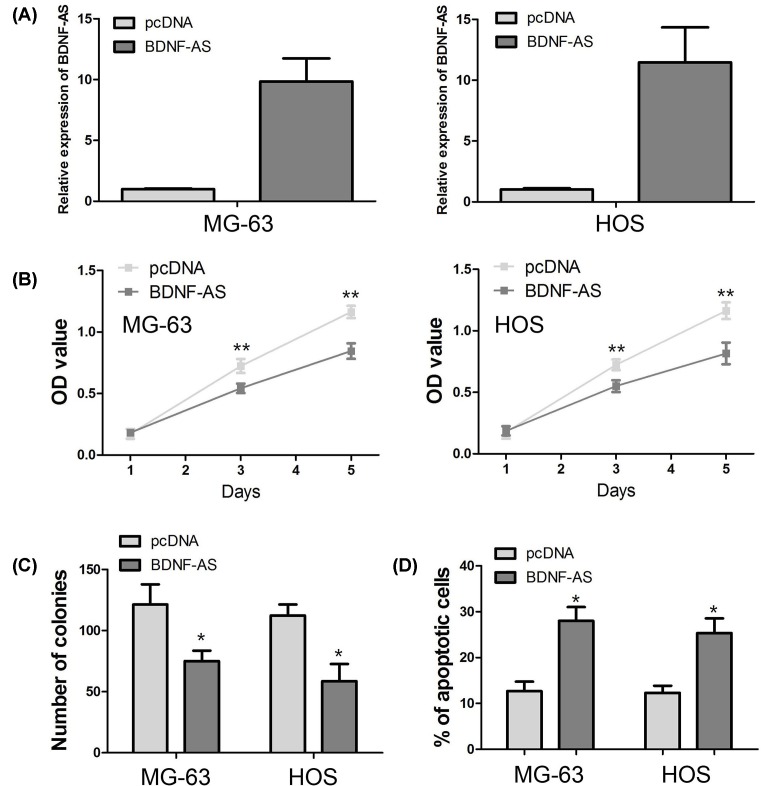
The biological function of BDNF-AS in OS cells (**A**) MG-63 and HOS cells are transfected with BDNF-AS overexpressing vector, and the transfection efficiency is confirmed by qRT-PCR. (**B**) BDNF-AS overexpression depresses the proliferation of MG-63 and HOS cells compared with their controls. (**C**) The number of colonies is decreased in BDNF-AS overexpressing OS cells compared with their controls. (**D**) The percentage of apoptotic cells in BDNF-AS overexpressing OS cells is higher than their controls. *P<0.05; **P<0.001

**Figure 3:**
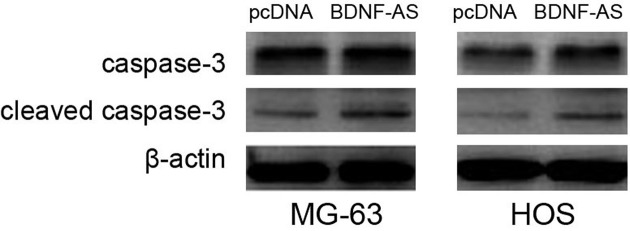
The effect of BDNF-AS on apoptosis-associated gene BDNF-AS overexpression increases cleaved caspase-3 protein expression in MG-63 and HOS cells, but had no effect on caspase-3.

Because above result suggested BDNF-AS expression was associated with tumor sizes in OS patients, the effects of BDNF-AS on cell proliferation and apoptosis *in vitro* were evaluated in OS cells. MG-63 and HOS cells were transfected with BDNF-AS overexpressing vector, and the transfection efficiency was confirmed by qRT-PCR (Figure 2A). Then, we evaluated cell proliferation in two OS cell lines using MTT assay and cell colony formation assay. After 72 and 120 h of incubation, BDNF-AS overexpression markedly depressed the proliferation of MG-63 and HOS cells compared with their controls (*P*<0.001, Figure 2B). Similarly, the number of colonies was obviously decreased in BDNF-AS overexpressing OS cells compared with their controls (*P*<0.05, Figure 2C). The results of apoptosis assay showed the percentage of apoptotic cells in BDNF-AS overexpressing OS cells was higher than their controls (*P*<0.01, Figure 2D). Subsequently, we measured apoptosis-associated gene caspase-3 and cleaved caspase-3 expression through Western blot, and observed BDNF-AS overexpression obviously increased cleaved caspase-3 protein expression in MG-63 and HOS cells, but had no effect on caspase-3 (Figure 3).

## Discussion

BDNF-AS is located on human chromosome 11p14.1. Originally, BDNF was discovered as a natural non-coding antisense of neuronal transcriptional factor BDNF to negatively regulate BDNF in neuronal system [14,15]. Recently, BDNF-AS has been suggested to be down-regulated in several types of human cancer such as lung cancer [16], prostate cancer [17], esophageal cancer [18], cervical cancer [19], and retinoblastoma [20]. However, the expression status of BDNF-AS is still unknown in OS. Thus, we detected the levels of BDNF-AS expression in pairs of OS tissues and non-cancerous normal tissues through qRT-PCR, found the expression of BDNF-AS was reduced in OS tissues. Meanwhile, we also observed that levels of BDNF-AS expression were decreased in OS cell lines compared with human normal osteoblast cell line hFOB1.19.

The clinical value of BDNF-AS was further estimated in our study. We analyzed correlations between BDNF-AS expression and clinicopathological parameters of OS cases, and found reduced BDNF-AS expression was correlated with advanced Enneking stage and large tumor size in OS patients. Similarly, Shen et al. [16] reported that low expression of BDNF-AS was significantly correlated with non-small cell lung cancer patients’ advanced clinical stage and lymph node metastasis. In prostate cancer patients, Li et al. [17] found that low levels of BDNF-AS were associated with advanced clinical stage high, more lymph node metastasis, and high Gleason score. Moreover, Shang et al. [20] indicated that reduced expression of BDNF-AS was associated with advanced clinical stage and poor differentiation in retinoblastoma cases.

The prognostic significance of BDNF-AS was investigated in lung cancer [16], prostate cancer [17], and retinoblastoma [20]. In non-small cell lung cancer patients, Shen et al. [16] showed patients with low expression of BDNF-AS had much worse overall survival than patients with high expression of BDNF-AS, and low expression of BDNF-AS served as an independent predictor for overall survival. Moreover, Li et al. [17] demonstrated that levels of BDNF-AS was positively associated with correlated with prostate cancer patients’ overall survival, and low BDNF-AS expression was an independent unfavorable factor. Besides, Shang et al. [20] reported BDNF-AS low expression was correlated with poor prognosis and served as an independent unfavorable marker for retinoblastoma patients’ overall survival. Similarly, we also found the low expression of BDNF-AS group had a significantly poorer prognosis than the high expression of BDNF-AS group, and BDNF-AS low expression was an independent unfavorable prognostic factor for overall survival in OS patients.

BDNF-AS has been suggested to function in tumor suppressive role in regulating tumor cell proliferation, apoptosis, migration, and invasion [16–20]. Meanwhile, our clinical study showed BDNF-AS expression was associated with tumor size in OS patients. Thus, we suppose that BDNF-AS also acts as a tumor suppressor to regulate OS cell proliferation and apoptosis. The gain-of-function study indicated that BDNF-AS overexpression inhibits OS cell proliferation and promotes cell apoptosis. In addition, BDNF-AS overexpression could induce neuronal cell apoptosis through regulating caspase-3 expression [21]. Thus, we further explored the effect of BDNF-AS on caspase-3 expression, and found BDNF-AS overexpression depressed the cleaved caspase-3 expression in OS cells.

In conclusion, BDNF-AS expression is decreased in OS tissues and cells. BDNF-AS low expression is correlated with advanced Enneking stage and large tumor size and poor prognosis in OS patients. BDNF-AS overexpression inhibits OS cell proliferation and promotes cell apoptosis through regulating cleaved caspase-3.

## Abbreviations

BDNF-AS: brain-derived neurotrophic factor antisense
lncRNA: long non-coding RNA
OS: osteosarcoma
qRT-PCR: quantitative real-time PCR
